# Preparation and Application of Phosphorylated Xylan as a Flocculant for Cationic Ethyl Violet Dye

**DOI:** 10.3390/polym10030317

**Published:** 2018-03-14

**Authors:** Zhongming Liu, Dingding Xu, Nannan Xia, Xin Zhao, Fangong Kong, Shoujuan Wang, Pedram Fatehi

**Affiliations:** 1 Key Laboratory of Pulp & Paper Science and Technology Ministry of Education, Qilu University of Technology (Shandong Academy of Sciences), Jinan 250353, China; liuzhongming126@126.com (Z.L.); 18364195258@163.com (D.X.); xianan00001@126.com (N.X.); zhaoxin_zixi@126.com (X.Z.); 2 Department of Chemical Engineering, Lakehead University, Thunder Bay, ON P7B 5E1, Canada; pfatehi@lakeheadu.ca

**Keywords:** xylan, phosphorylated xylan, dye removal, flocculation

## Abstract

In this study, phosphorylated birchwood xylan was produced under alkali conditions using trisodium trimetaphosphate. Three single-factor experiments were used to explore the influences of time, temperature, and the molar ratio of trisodium trimetaphosphate to xylan on the degree of substitution (DS) and charge density of xylan. The response surface methodology was used to explore the interaction of these three factors. Phosphorylated xylan with a maximum DS of 0.79 and a charge density of −3.40 mmol/g was produced under the optimal conditions of 80 °C, 4 h, and a molar ratio of xylan/sodium trimetaphosphate (STMP) of 1/3. Fourier transform infrared (FTIR), ascorbic acid method analyses, and inductively coupled plasma–atomic emission spectrometer (ICP-AES) analyses confirmed that the phosphate groups were successfully attached to xylan. Thermogravimetric analysis confirmed that phosphorylated xylan was less stable than birchwood xylan. Furthermore, the phosphorylated xylan was applied as a flocculant for removing ethyl violet dye from a simulated dye solution. The results indicated that more than 95% of the dye was removed from the solution. The theoretical and experimental values of charge neutralization for the dye removal were close to one another, confirming that charge neutralization was the main mechanism for the interaction of dye and phosphorylated xylan. The impacts of salts on the flocculation efficiency of phosphorylated xylan were also analyzed.

## 1. Introduction

Xylan is an abundant natural polymer with a structure similar to that of cellulose; it accounts for 15–30% of the biomass [1,2]. The polymeric backbone of xylan is composed of repeating xylose units that are connected by a β-1-4 linkage [3]. Functional groups attached to xylan can assist its modification, which helps with the application of modified xylan as a value-added product such as medicines [4,5], biomaterials [6], hydrocolloids [7], micro-particles [8], and adsorbents for wastewater [9]. A cationic xylan copolymer has also been claimed to be effective as a flocculant for removing dyes from wastewater in the textile industry [10].

Ethyl violet (EV) is a triphenylmethane dye with extensive industrial applications [11]. It has been reported that a significant amount of the dye could be lost during the dyeing processes of the textile industry and thus released to the wastewater [11]. Therefore, the removal of the dye from wastewater is crucial for improving the environmental impact of the textile industry. Several chemical and physical methods have been developed to remove synthetic dyes from wastewater such as incineration, ozonation, adsorption, coagulation, electrochemical oxidation, and using membranes [12]. Despite a high efficiency in dye removal, the complexity and costs of the recycling for adsorbents in adsorption processes may be prohibitive to some applications. Phosphorylation has been one of the most effective chemical-modification techniques for introducing phosphate groups to polymers that have –OH functional groups to produce, for instance, adsorption materials [13,14] and hydrogels [15].

Among the modification methods, phosphorylation modification methods can be used to significantly enhance bioactivities [16], which comprise good antitumor, antioxidant, and antiviral activities [17]. In one study, a phosphorylated *Codonopsis pilosula* polysaccharide was synthesized and characterized, and its ability to inhibit the virulence of duck hepatitis A virus was compared with that of an unmodified *Codonopsis pilosula* polysaccharide [17]. In another study, a phosphorylated sago starch-extraction residue was prepared for the removal of cadmium from wastewater [14]. Song and coworkers produced a phosphorylated pumpkin polysaccharide with antioxidant activities [18]. Ghimici and Suflet were successful in producing phosphorylated polysaccharide derivatives as efficient separation agents for zinc and ferric-oxide particles from water [19]. Ming and Wang produced the phosphorylated *Chrysanthemum indicum* polysaccharide with a degree of substitution (DS) of 3.17 using the STMP-STPP (sodium trimetaphosphate, sodium tripolyphosphate) method [20]. However, the preparation of phosphorylated xylan and its application as a flocculant for removing dyes from solutions have not been reported and is, in fact, the objective of this work.

The current study investigated the production of phosphorylated xylan with STMP and the interaction of reaction parameters to obtain phosphorylated xylan with a high DS and charge density under alkali conditions. Furthermore, the characteristics of the prepared phosphorylated xylan and the application of phosphorylated xylan as a flocculant for the removal of ethyl violet dye from a simulated dye solution were analyzed. The main novelties of this work are the following: (1) the production of phosphorylated xylan and the optimization of the phosphorylation reaction of xylan by grafting STMP onto a xylan backbone; and (2) the assessment of the performance of phosphorylated xylan as a flocculant in isolating cationic dye segments from a simulated ethyl violet dye solution.

## 2. Materials and Methods 

### 2.1. Materials

**Figure 1:**
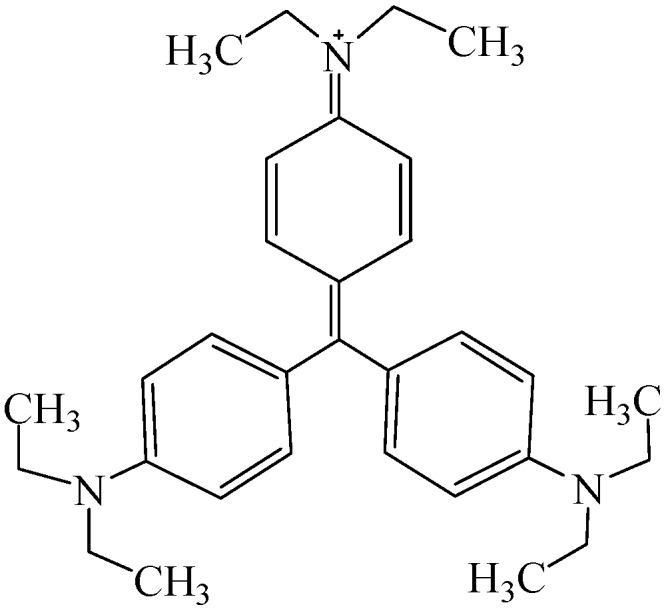
The chemical structure of cationic ethyl violet dye.

Xylan from birchwood, STMP used as received without further purification, and polydiallyldimethylammonium chloride (PDADMAC) diluted to 0.001 M prior to use were purchased from Sigma-Aldrich, Shanghai, China. EV dye was also obtained from Sigma Aldrich and diluted to 100 mg/L for use. All other chemicals used herein were of analytical grade and without further purification. The chemical structure of ethyl violet is presented in Figure 1. It is clear that the quaternary ammonium group contributes to the cationic charge of the dye. The cationic charge of the dye reduces to one mol per dye segment at a high pH due to the interaction of the hydroxyl group with the quaternary ammonium group and the deprotonation of the ternary ammonium group of the dye. At a very low pH, the dye segment may have up to 3 moles of charged group on its segment in 1 mole dye. 

### 2.2. Phosphorylation of Xylan

The phosphorylation procedure of xylan was carried out using STMP as a crosslinker, according to the method stated earlier [15,21]. Birch xylan (1.32 g, 10 mmol) was mixed with 45 mL of deionized water in a closed container, STMP (10 mmol, 20 mmol, 30 mmol, 40 mmol, and 50 mmol) as a crosslinking reagent was added into the solution, 1 mol/L sodium hydroxide was used to adjust the pH to 9 under constant stirring (150 rpm), and the reaction occurred at a certain temperature (50 °C, 60 °C, 70 °C, 80 °C and 90 °C) for 2, 3, 4, 5, or 6 h. Upon completion, the solution was cooled to room temperature and neutralized with a 1 mol/L hydrochloric acid solution. Next, the solution was dropped into 200 mL of 95% ethanol solution. The precipitate was then washed twice with 200 mL of 95% ethanol and dialyzed with the membrane dialysis for 24 h against deionized water. Finally, the dialyzed samples were dried in a blast oven to obtain purified phosphorylated xylan. In this set of experiments, the reaction was carried out at one variable parameter while the other condition was fixed, one-factor experiments. The DS and charge density of the phosphorylated xylan was used as the response of molar ratio of xylan to STMP, reaction time, and temperature to the reaction. All the experiments were repeated three times, and the average values reported in this work.

**Figure 2:**
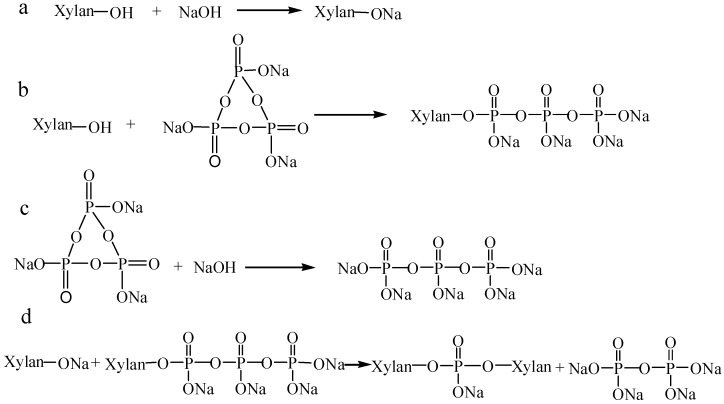
The reaction of xylan and sodium trimetaphosphate (STMP).

The reaction mechanism of xylan with STMP (as a crosslinking agent) involved in this study was a reaction between the hydroxyl groups in the xylan and metaphosphate groups in STMP, leading to phosphate ester (O–P–O) linkages between two polysaccharide moieties to produce phosphorylated xylan [21,22,23], as shown in Figure 2.

### 2.3. Experimental Design

Response surface methodology (RSM) is a powerful technique that can be applied to explore the interactions between factors in experiments and to optimize the operating conditions to obtain the best results [24]. To explore the interaction of reaction time, temperature, and the ratio of xylan/STMP in the xylan phosphorylation, Design-Expert Version 8.0.5 software (State-Ease, MN, USA) was used. In this set of experiments, three factors and three levels were considered in 17 runs according to the Box–Behnken method. The factors and levels are listed in Table 1. The DS and charge density of phosphorylated xylan were used as responses of the factors to the reaction. Five replicated experiments were used to evaluate and reduce measurement errors. The experiments were repeated three times, and the average values reported in this work.

### 2.4. Analytical Methods

#### 2.4.1. Fourier Transform Infrared (FTIR) Analysis

FTIR analysis was conducted on the xylan and phosphorylated xylan samples using a FTIR spectrophotometer (Bruker VERTEX70, Rheinstetten, Germany). In this measurement, unmodified and phosphorylated xylan samples of 0.01 g each were used. Each spectrum was recorded with 32 scans in transmittance mode with a resolution of 0.5 cm^−1^ within the range 400 to 4000 cm^−1^.

#### 2.4.2. Charge Density Analysis

Approximately unmodified and phosphorylated xylan samples of 0.02 g each were dissolved in 100 mL of deionized water and ultrasounded at 30 °C for 1 h. The solution was used to measure charge density using a particle-charge detector (Mutek, PCD 03, Herrsching, Germany) against a 0.001 M PDADMAC standard solution.

#### 2.4.3. Determination of Phosphorus Content and Degree of Substitution (DS)

The phosphate radical content of the phosphorylated xylan was determined using the ascorbic-acid method [20]. According to the established formula deduced from the expression for the phosphorus content ratio, Equations (1) and (2) for calculating DS can be obtained as follows.

(1) $$P\%\;=\;\frac{DS\times 3b\times 100}{\left(a+DS\times c\right)}\;$$

(2) $$\mathrm{DS}=\frac{a\times P\%}{300b-c\times P\%}\;=\frac{132\times P\%}{300\times 31-124\times P\%}=\frac{1.32\times P\%}{93-1.24\times P\%}\;$$

where *P*% represents the phosphorus content; *a* is the molecular weights of the unmodified xylan’s monomeric unit, 132 g/mol; *b* is the mass of phosphorous atom, 31 g/mol; and *c* is the added molecular weight when –OH in xylan was substituted by –OPO_3_Na_2_ [25], 124 g/mol.

#### 2.4.4. Molecular Weight Analysis 

Dried unmodified and phosphorylated xylan samples of approximately 5 mg each were dissolved in 0.1 mol/L NaNO_3_ by stirring at 500 rpm for 36 h at 35 °C, and the solutions were then filtered using a 0.2 µm nylon filter. The filtered solutions were used for molecular weight analysis. The molecular weight analysis of the samples was carried out using gel permeation chromatography, Heleos-II GPC (Wyatt Technology, Santa Barbara, CA, USA) with a multi-angle laser light scattering detector. The columns of PolyAnalytic PAA 206 and PAA 203 were set up at 35 °C, and a 0.1 mol/L NaNO_3_ solution was used as a solvent and an eluent. The flow rate was set at 0.50 mL/min, while poly(ethylene oxide)s were used as standard samples for the calibration of this aqueous system. The degree of polymerization of unmodified and modified xylan was calculated based on Equation (3).

(3) $$\mathrm{Degree}\;\mathrm{of}\;\mathrm{polymerization}\;=\;\frac{M_{n}}{132+DS\times 124}\;$$

where *M_n_* is the number averaged molecular weight of xylan, g/mol. The molecular weight of xylan’s monomeric unit is 132 g/mol. *DS* is the degree of substitution of modified xylan. The added molecular weight when –OH in xylan was substituted by –OPO_3_Na_2_ is 124 g/mol.

#### 2.4.5. Elemental Analysis

Elemental analysis of phosphorus in xylan and phosphorylated xylan was carried out using an inductively coupled plasma–atomic emission spectrometer (ICP-AES; ICAP 9000, Shimadzu, Tokyo, Japan). The sample was dissolved in 0.1 mol/L HCl and further diluted with water. The measured concentration of phosphorus in aqueous solution was converted into actual phosphorus content, taking into consideration the dilution factor [26]. The contents of carbon (C), hydrogen (H), and oxygen (O) in the xylan and phosphorylated xylan were measured with an elemental analyzer (Vario EL III, Elementar Analysensysteme, Hanau, Germany). The samples were combusted at up to 900 °C to 1200 °C for analytical accuracy with a large dynamic range of elemental concentration, e.g., up to 30 mg of carbon absolute. 

#### 2.4.6. Thermogravimetric Analysis

The thermal analysis of unmodified and phosphorylated xylan samples was performed on a thermogravimetric analyzer (TGA Q50, New Castle, DE, USA). Samples of 3–10 mg were heated from room temperature to 600 °C at the rate of 10 °C/min under a nitrogen environment to examine the thermal stability properties.

#### 2.4.7. Viscosity Measurement

The viscosities of xylan and phosphorylated xylan were measured at different concentrations at 25 °C using a Brookfield DV-II + Pro viscometer (Brookfield Engineering Laboratory, Inc., Middlesboro, MA, USA). The viscosities were measured in aqueous solutions (pH 7) at room temperature. In this set of experiments, different concentrations of xylan or phosphorylated xylan solutions were placed in the spindle No. S61 of the viscometer, and the spindle rotation was adjusted from 1 up to 100 rpm to measure the viscosity of the samples.

#### 2.4.8. Flocculation Analysis

In this set of experiments, ethyl violet dye solutions were prepared at different concentrations by dissolving the dye in deionized water, and the pH of the dye solutions was adjusted to the desired value by adding a HCl or NaOH solution to it. Then, different amounts of phosphorylated xylan solution were added to the dye solutions, and the mixtures were continuously stirred at 30 °C for 30 min at 150 rpm. After that, the mixtures were centrifuged at 10,000 rpm for 10 min, and the filtrate was collected for analysis. The concentration of the dye in the solutions (before mixing with phosphorylated xylan and after centrifugation) was measured using a UV-2550 spectrophotometer (Shimadzu Co, Kyoto, Japan) at a wavelength of 596 nm. The removal of the dye from the solution was calculated according to the following equation:

(4) $$\mathrm{Dye}\;\mathrm{removal}=\frac{C_{0}-C}{C_{0}}\times 100\%$$

where *C*_0_ refers to the concentration of the ethyl violet dye in the control sample (mg/L); and *C* refers to the concentration of the dye after treatment with phosphorylated xylan (mg/L). To investigate the impact of salts on the efficiency of phosphorylated xylan in removing the dye from the solution, dye solutions (100 mg/L) containing 0.01 mol/L NaCl, NaNO_3_, and FeSO_4_ were prepared. Phosphorylated xylan was added to 30 mL of a dye solution containing salts at pH 9. After that, the dye solution was treated as stated above (the mixtures were centrifuged at 10,000 rpm for 10 min, and the filtrate was collected for analysis), and the removal of the dye was determined according to Equation (4). The experiments were repeated three times, and the average values reported in this work.

#### 2.4.9. COD Analysis

The chemical oxygen demand (COD) test is commonly used to indirectly measure the amount of organic pollutants found in wastewater. In this set of experiments, the samples were treated with potassium dichromate under acidic conditions with silver as a catalyst at 150 °C for 2 h in a thermoreactor (CR 2200, WTW GmbH, Weilheim, Germany). The solutions were cooled for 20 min in the digester then stored in the dark for 30 min [27]. The concentration of the COD in the solutions (before and after mixing with phosphorylated xylan) was measured using the UV-2550 spectrophotometer at the wavelength of 620 nm. The COD removal was calculated according to Equation (5):

(5) $$\mathrm{COD}\;\mathrm{removal}=\frac{C_{o}-C}{C_{o}}\times 100\%$$

where *C*_o_ and *C* are the COD concentrations of the dye solutions before and after treating with phosphorylated xylan, mg/L, respectively.

## 3. Results

### 3.1. Influences of Reaction Conditions on DS and Charge Density of Phosphorylated Xylan

#### 3.1.1. Molar Ratio of Xylan/STMP

**Figure 3:**
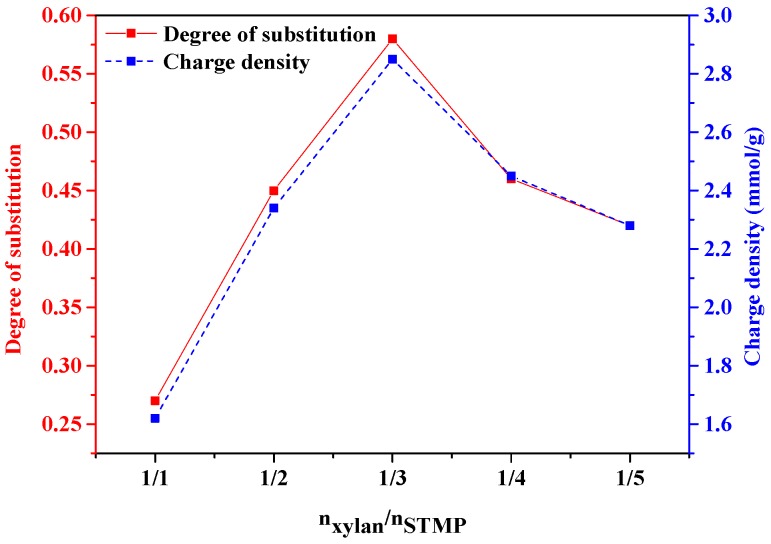
The effect of molar ratio of xylan/STMP (n_xylan_/n_STMP_) on the degree of substitution and charge density of phosphorylated xylan (conducted at 80 °C for 3 h).

Figure 3 illustrates the impact of the xylan/STMP ratio on the DS and charge density of phosphorylated xylan. The DS and charge density increased when the molar ratio was increased from 1/1 to 1/3, and a higher than molar ratio of 1/3 insignificantly changed the DS and charge density. The results confirmed that the increase in the concentration of STMP enhanced the DS and charge density of the produced phosphorylated xylan; this may be due to the increase in the collision chances of chemical agents at higher concentrations in the reaction media. It was also noted that the highest molar ratio of 1/5 did not improve the properties of phosphorylated xylan; this may be attributed to the progress in side reactions (Figure 2c). This phenomenon has also been claimed in the past in the preparation of Pullulan–STMP hydrogels [23]. Therefore, the molar ratio of 1/3 for xylan/STMP was selected for further analysis.

#### 3.1.2. Temperature

**Figure 4:**
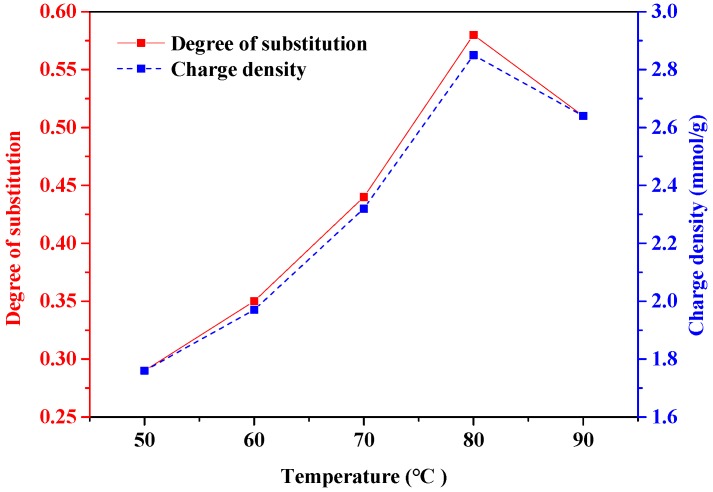
The effect of temperature on the degree of substitution and charge density of phosphorylated xylan (conducted at the xylan/STMP molar ratio of 1/3 for 3 h).

The effect of the reaction temperature on the DS and charge density of phosphorylated xylan is illustrated in Figure 4. It is evident that, with the increase in the reaction temperature from 50 °C to 80 °C, the DS and charge density of phosphorylated xylan increased to 0.58 and −2.85 mmol/g, respectively, due to favorable conditions for phosphorylation. However, further temperature increases to 90 °C slightly reduced the DS and the charge density of phosphorylated xylan to 0.63 and −2.64 mmol/g, respectively. The decrease in the DS and charge density at a temperature higher than 80 °C was due to the degradation of xylan and the generation of undesired products as stated in previous research [24].

#### 3.1.3. Time

**Figure 5:**
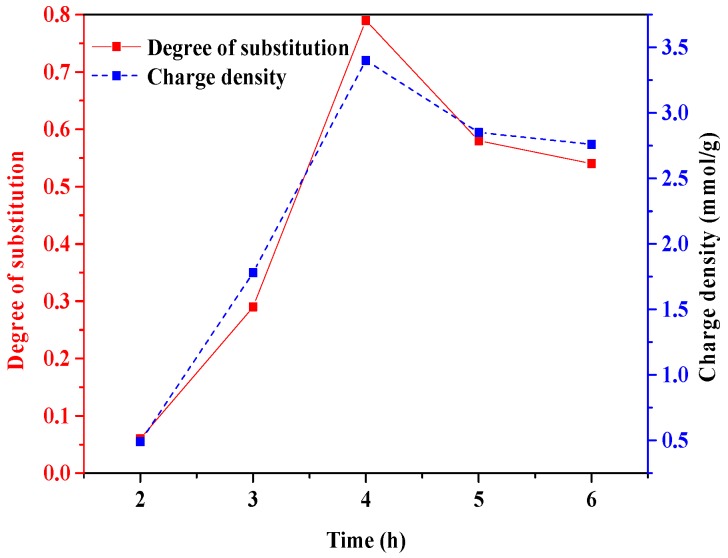
The effect of time on the degree of substitution and charge density of phosphorylated xylan (conducted at the xylan/STMP molar ratio of 1/3 and 80 °C).

Figure 5 represents the influence of reaction time on the DS and charge density of phosphorylated xylan. The maximum DS of 0.79 and charge density of −3.40 mmol/g were obtained at 4 h. There was an increase in the DS and charge density of phosphorylated xylan when time was increased from 2 h to 4 h, after which they decreased. However, when the time was longer than 4 h, the DS and charge density of phosphorylated xylan decreased due to the increase of the undesired product (sodium tripolyphosphate and pyrophosphate) hindering the production of phosphorylated xylan [24].

### 3.2. Analysis of Variance (ANOVA)

The results in Figure 3, Figure 4 and Figure 5 do not show which reaction parameters had the most significant impact on the charge density and DS of the phosphorylated xylan. The relationship between the experimental factors and the generated results as well as the contribution of experimental factors can be determined by the ANOVA statistical technique. To further understand the impact of process parameters on the efficiency of reaction and to be able to predict the phosphorylation reaction of xylan, the outcomes of the modeling analysis in Table 1 (i.e., sum of squares, degrees of freedom, mean square, *F*-value, and *p*-values) were estimated and are presented in Table 2. In the case of *p*-value, A, B, C, AB, AC, BC, A^2^, B^2^, and C^2^ were less than 0.05, implying that the correlation factors were significant [28]. The predicted R^2^ of 0.9790 and 0.9395 for DS and charge density were in agreement with the adjusted R^2^ of 0.9970 and 0.9914, respectively. In this case, the F-value of B (Temperature) was the smallest of the three factors, indicating the negligible effect of temperature when compared to other factors (time and xylan/STMP molar ratio) in increasing the DS and charge density of phosphorylated xylan. Furthermore, it was clear that time had a major effect on the DS and charge density of phosphorylated xylan [24]. 

### 3.3. Properties of Phosphorylated Xylan with the Maximum DS and Charge Density

The properties of phosphorylated xylan with a DS of 0.79 and charge density of −3.40 mmol/g, which were produced under the conditions of 80 °C, 4 h, and with a xylan/STMP molar ratio of 1/3, are listed in Table 3. The weight-average molecular weight (*Mw*) and number-average molecular weight (*Mn*) of the phosphorylated xylan sample were 23,500 g/mol and 18,600 g/mol, respectively, whereas those of unmodified xylan were 20,800 g/mol and 11,850 g/mol, respectively. A slight increase in the molecular weight of xylan due to the grafting phosphate group was also observed in another work [29]. The decrease in the polydispersity, the ratio of *Mw* to *Mn*, may be due to the grafting of the phosphate group to xylan. The degree of polymerization of xylan was decreased from 90 to 81, which was probably due to the degradation of the xylan chain in alkaline conditions [30].

### 3.4. Elemental Analysis

Elemental analysis of the xylan and phosphorylated xylan was carried out to determine the content of carbon, hydrogen, oxygen, and phosphorous in xylan and phosphorylated xylan and the data is listed in Table 4. The C_5_ unit formulas of xylan and phosphorylated xylan were also calculated based on the elemental analysis. It was clear that the carbon content decreased from 42.5 to 19.1%, the oxygen content increased from 47.1 to 55.9%, and the hydrogen content decreased from 5.8 to 2.21%, which was attributed to the high content of oxygen and the low content of carbon and no hydrogen in –OPO_3_Na_2_ grafted to xylan backbone. The content of phosphorus increased from 0.03% of unmodified xylan to 23.10% of phosphorylated xylan. The phosphorus here was clearly from the –OPO_3_Na_2_ segment in phosphorylated xylan as the unmodified xylan only had unnoticeable phosphorus content (0.03 wt %). These changes confirmed the successful phosphorylation of xylan using STMP. In addition, the phosphorous content measured using ICP-AES, 31.81%, was used to calculate the DS through Equation (1), and was 0.783, which was very close to the 0.79 value obtained using the ascorbic-acid method.

### 3.5. FTIR Analysis

**Figure 6:**
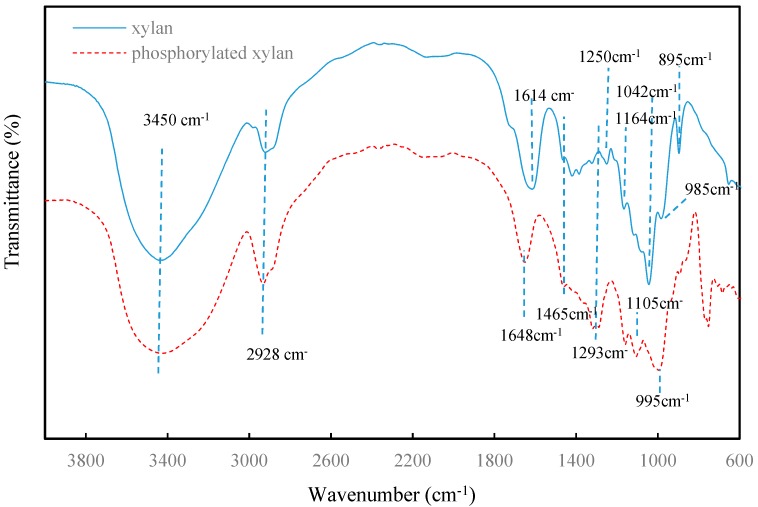
FTIR spectra of xylan and phosphorylated xylan.

FTIR spectra of unmodified and phosphorylated xylan are illustrated in Figure 6. The absorption peaks at 3427, 2928, 1648, 1465, 1250, 1164, 1042, 985, and 895 cm^−1^ were associated with xylan. A broad absorbance peak at 3427 cm^−1^ corresponded to the hydroxyl stretching vibration [31]. The absorption peak at 2928 cm^−1^ was assigned to the symmetric C–H stretching vibration, and that at 1648 cm^−1^ contributed to the absorbed water in the samples [32]. The strong peak at 1042 cm^−1^ was assigned to C–O stretching in C–O–C linkages [33,34]. The sharp peak at 895 cm^−1^ was assigned to the β-glucosidic linkage between the xylose units, indicating that the xylose residues were linked by β-form bonds [35]. The low intensity of the bands at 985 cm^−1^ and 1164 cm^−1^ suggested the presence of arabinosyl units, which were only attached at position 3 of the xylopyranosyl constituents [35]. The region between 1465 cm^−1^ and 1042 cm^−1^ related to the C–H and C–O bond stretching frequencies. Compared with the spectrum of xylan, the spectrum of phosphorylated xylan appeared to have three new peaks at 1293 cm^−1^, 1105 cm^−1^, and 995 cm^−1^, respectively. The new adsorption peak at 1293 cm^−1^ was ascribed to the stretching vibration of the P=O band. The peak at 1105 cm^−1^ originated from the stretching vibration of the C–O–P band [14,17]. The peak at 995 cm^−1^ was ascribed to the O–P–O band [36]. These new peaks confirmed that the phosphorylated xylan was successfully synthesized.

### 3.6. Thermogravimetric Analysis

**Figure 7:**
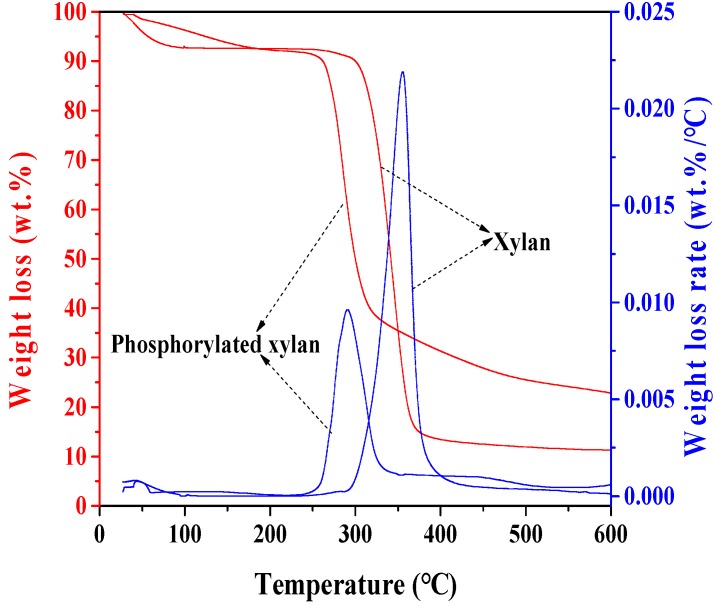
Weight loss and weight loss rate of xylan and phosphorylated xylan.

Thermal analysis of unmodified xylan and phosphorylated xylan are illustrated in Figure 7. The samples of unmodified xylan and phosphorylated xylan were analyzed at a heating rate of 10 °C/min under a nitrogen environment. The weight of unmodified xylan and phosphorylated xylan decreased gradually with the increase in the temperature from room temperature to 150 °C; this was attributed to the loss of moisture in the samples [37]. The weight loss of unmodified xylan started at around 230 °C while that of phosphorylated xylan started at 210 °C. The main degradation temperature of xylan ranged from 250 °C to 450 °C; however, that of phosphorylated xylan ranged from 240 °C to 350 °C; this suggests that the chemical modification led to the lower thermal stability of the final products [27]. When the temperature reached 400 °C, only 11 wt % of the xylan sample was left. However, at this temperature, more than 20 wt % of phosphorylated xylan was left, which was mainly due to the phosphorylated groups as stated in previous works [13].

### 3.7. Viscosity Analysis of Xylan and Phophorylated Xylan Solutions

**Figure 8:**
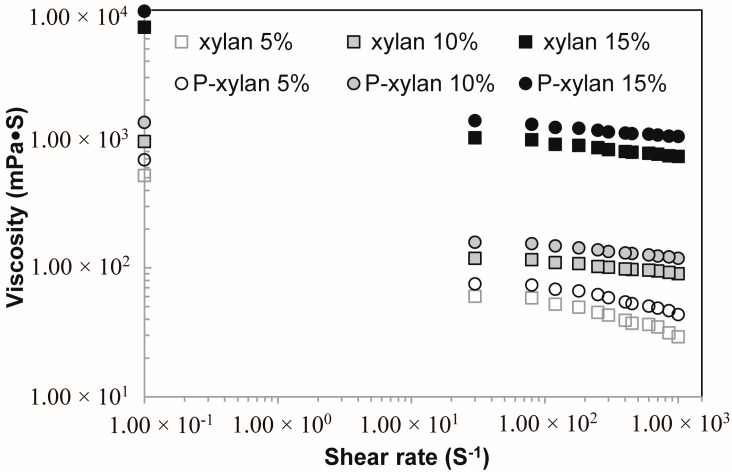
Viscosity of xylan and phosphorylated xylan (P-xylan) solutions as function of shear rate at various concentration.

Figure 8 shows the dynamic viscosity of xylan and phosphorylated xylan solutions at concentrations of 5%, 10%, and 15% (*w*/*w*). The viscosity of xylan and phosphorylated xylan solutions decreased with increasing shear rate at the same concentration, exhibiting a pseudoplastic or shear-thinning behavior in the range of shear rates tested. Figure 8 also indicates that the viscosity of the xylan and phosphorylated xylan solutions increased when the concentration was increased. This was probably due to the stronger entanglements between xylan molecules at a higher concentration. When the shear rate increases, this network structure is destroyed; therefore, a shear thinning behavior is observed [38,39]. It was also found that the phosphorylated xylan showed a higher viscosity than unmodified xylan, which was probably due to the higher molecular weight and stronger intermolecular interactions between the phosphorylated xylan molecules. 

### 3.8. Flocculation Analysis

#### 3.8.1. The Effect of Dye Concentrations and Phosphorylated Xylan Concentrations on Dye Removal

**Figure 9:**
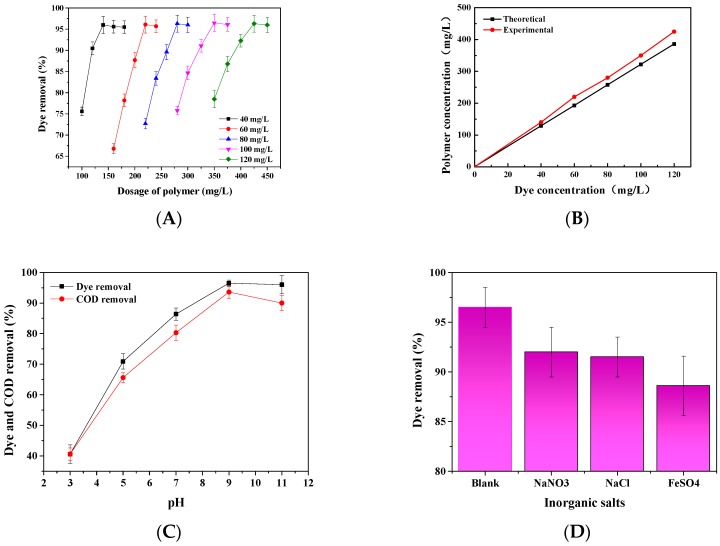
(**A**) The effect of initial dye and phosphorylated xylan concentrations on dye removal under conditions of 30 °C and pH 9; (**B**) the relationship between optimum and theoretical phosphorylated xylan dosage and initial concentration; (**C**) the effect of pH on dye removal under conditions of 100 mg/L dye concentration, 350 mg/L phosphorylated xylan concentration and 30 °C; and (**D**) the effect of inorganic salts on dye removal under the conditions of 100 mg/L dye concentration, 350 mg/L phosphorylated xylan concentration, pH 9, and 30 °C.

The effect of dye concentration on the efficiency of phosphorylated xylan is illustrated in Figure 9A. The dye concentration was varied between 40 and 120 mg/L so that the optimum concentration of phosphorylated xylan for removing dyes with different concentrations could be determined. As expected, with an increase in the concentration of dye, the optimum phosphorylated xylan concentration for dye removal also increased. This indicated that the initial concentration of dye was an important factor in the dye’s interaction with phosphorylated xylan [40].

Based on the results in Figure 9A, the correlation between the optimum concentration of phosphorylated xylan (i.e., the concentration that generated the maximum dye removal in Figure 9A) and the initial dye concentrations is illustrated in Figure 9B. Based on the charge densities of phosphorylated xylan and dye and by considering the charge neutralization mechanism, the concentration of phosphorylated xylan required for achieving the maximum dye removal can be calculated theoretically. The theoretical concentration of phosphorylated xylan needed to achieve the maximum dye removal at different dye concentrations is depicted in Figure 9B. When the concentration of dye was low, the theoretical and experimental values were close, confirming the importance of charge neutralization. When the concentration of the dye was high, the concentration required experimentally was much higher than that required theoretically [27]. This is because not all of the charges on dye or phosphorylated xylan are available for interaction, and this is more observable at higher dye concentrations that have stronger charge repulsion from adjacent polyelectrolytes [27,41]. The deviation seen in Figure 9B may be attributed to: (1) experimental errors; (2) unstoichiometric charge interaction of phosphorylated xylan and the dye segment; as well as (3) hydrogen bonding development between the dye and phosphorylated xylan in the solution.

#### 3.8.2. The Effect of pH on Dye and COD Removal

Figure 9C demonstrates the effect of pH on the dye and COD removals. As expected, the dye removal was almost complete under alkaline conditions, pH 9, and were insignificantly changed by increasing pH from 9 to 11, but COD removal slightly decreased. This may indicate that phosphorylated xylan was more effectively involved in the flocculant formation with the dye segments at a pH higher than 9. Under an acidic pH, free protons would interact with phosphorylated xylan, which would reduce their efficiency [42]. As stated earlier, the cationic charge density of dye is reduced at a high pH, which hampers the dye’s water solubility. The reduced charge density also implies that the minimum amount of phosphorylated xylan required for efficient dye removal based on the charge neutralization is reduced. At a high pH of 11, the hydroxyl group may interact with the dye segments, reducing the attractive force developed between the phosphorylated xylan and dye for flocculant formation [43].

#### 3.8.3. Inorganic Salts

Salts are generally available in dyeing wastewater and may affect the efficiency of phosphorylated xylan in dye removals from the solution. Figure 9D illustrates the impact of various inorganic salts (0.01 mol/L concentration) on dye removals using phosphorylated xylan. In general, the addition of various inorganic salts slightly reduced the efficiency of phosphorylated xylan in removing dyes from the solutions. Ferrous sulfate reduced the dye removal by 10%, which was consistent with previously reported results [27]. When the monovalent Na^+^ cation is added to the dye solution, it generates the electrolyte effect, which reduces the dye removal efficiency of phosphorylated xylan. If multivalent cations such as Fe^2+^ are present in the dye solution, there is competition between the cation and the dye segments to bind to the anionic sites of the phosphorylated xylan. These chelated units exert a partially positive charge, which promotes electrostatic repulsion between the phosphorylated xylan and the dye in the solutions [44].

### 3.9. Industrial Applicability

This study indicates the effective interaction between phosphorylated xylan and a cationic dye. It is worth mentioning that the cationic dye was used only as a model of small molecules for the fundamental investigation in the current study. However, the practical application of phosphorylated xylan for dye removals may not be considered to be a stand-alone process since this process may generate phosphorylated xylan/dye sludges with a high water content that may be challenging to dispose of at industrial scales [12]. Therefore, a new separation process should be developed for the isolation of phosphorylated xylan/dye complexes to develop a phosphorylated xylan-based coagulation system for dye removal.

Simultaneous adsorption and flocculation have been reported to be effective in removing organic components from wastewater [45,46]. Phosphorylated xylan may be used along with other adsorbents for improving the efficiency of adsorbents in dye removal processes. The use of a coagulant in this case may help reduce the amounts of adsorbents used for dye removal, and a higher dye removal efficiency may be obtained if an adsorbent and a coagulant are used together. However, further studies are required to prove this concept, which was out of the scope of this study.

## 4. Conclusions

In this study, phosphorylated xylan with the maximum DS of 0.79 and charge density of −3.40 mmol/g was produced under the optimal conditions of 80 °C, 4 h, and a xylan/STMP molar ratio of 1/3. This product had a molecular weight (*Mw*) of 23,500 g/mol. FTIR, ascorbic acid analyses, and ICP-AES analyses confirmed that the phosphate groups were successfully grafted to the xylan backbone. The thermal stability of xylan was reduced through phosphorylation. The phosphorylated xylan was used as a flocculant, and the results confirmed that 96.5% of the dye and 93.6% of COD were removed from the dye solution (100 mg/L) with phosphorylated xylan at a concentration of 350 mg/L. Furthermore, phosphorylated xylan was most effective in dye removal at pH 9 and 30 °C. The experimental and theoretical analyses confirmed that the charge neutralization was the main interaction mechanism between the dye and phosphorylated xylan. The presence of salt slightly hampered the effectiveness of phosphorylated xylan in dye removal.

## Figures and Tables

**Table 1:** The parameters and outcomes of phosphorylation of xylan.

Run	Xylan/STMP, mol/mol	Temperature, °C	Time, h	Phosphorus Content, %	DS	Charge Density, mmol/g
1	1/3	70.00	4.00	10.41	0.76	3.30
2	1/3	70.00	4.00	10.41	0.76	3.30
3	1/3	70.00	4.00	10.41	0.76	3.30
4	1/5	70.00	2.00	2.53	0.12	0.92
5	1/3	70.00	4.00	10.41	0.76	3.30
6	1/1	50.00	4.00	5.21	0.28	1.62
7	1/3	50.00	6.00	5.64	0.31	1.81
8	1/5	90.00	4.00	8.82	0.58	2.85
9	1/3	90.00	2.00	1.75	0.08	0.65
10	1/3	70.00	4.00	10.41	0.76	3.30
11	1/1	90.00	4.00	5.06	0.27	1.62
12	1/5	70.00	6.00	8.41	0.54	2.76
13	1/3	90.00	6.00	8.10	0.51	2.64
14	1/1	70.00	2.00	1.33	0.06	0.49
15	1/1	70.00	6.00	4.44	0.23	1.45
16	1/5	50.00	4.00	6.19	0.35	1.97
17	1/3	50.00	2.00	0.91	0.04	0.32

**Table 2:** ANOVA analysis for degree of substitute (DS) and charge density of phosphorylated xylan.

Source	Sum of Squares		Mean Square		*F*-Value		*p*-Value	
	DS	Charge Density, mmol/g	DS	Charge Density, mmol/g	DS	Charge Density, mmol/g	DS	Charge Density, mmol/g
Model	1.20	18.66	0.13	2.07	592.23	204.97	0.0001	0.0001
xylan/STMP (A)	0.070	1.38	0.070	1.38	312.50	136.22	0.0001	0.0001
Temperature (B)	0.026	0.52	0.026	0.52	117.56	51.43	0.0001	0.0002
Time (C)	0.21	4.93	0.21	4.93	924.50	487.41	0.0001	0.0001
AB	0.014	0.19	0.014	0.19	64.00	19.14	0.0001	0.0033
AC	0.016	0.19	0.016	0.19	69.44	19.14	0.0001	0.0033
BC	0.006	0.063	0.006	0.063	28.44	6.18	0.0011	0.0419
A^2^	0.16	1.61	0.16	1.61	702.49	158.74	0.0001	0.0001
B^2^	0.16	1.88	0.16	1.88	720.73	185.48	0.0001	0.0001
C^2^	0.46	6.87	0.46	6.87	2022.49	679.40	0.0001	0.0001

**Table 3:** The properties of unmodified xylan and phosphorylated xylan.

Samples	Charge Density (mmol/g)	*Mw* (g/mol)	*Mn* (g/mol)	*Mw*/*Mn*	Degree of Polymerization
Unmodified Xylan	−0.12	20,800	11,850	1.76	90
Phosphorylated Xylan	−3.40	23,500	18,600	1.26	81

**Table 4:** The elemental analysis of xylan and phosphorylated xylan.

Samples	P, wt %	C, wt %	H, wt %	O, wt %	Formula
Xylan	0.03	42.50	5.80	47.10	C_5_H_8.22_O_4.13_P_0.001_
Phosphorylated xylan	31.81	26.22	3.15	70.90	C_5_H_7.21_O_10.13_P_2.34_
